# THE IMPACT OF HEALTH-RELATED QUALITY OF LIFE ON CLINICAL OUTCOMES IN ATRIAL FIBRILLATION POPULATIONS

**DOI:** 10.1093/geroni/igad104.2935

**Published:** 2023-12-21

**Authors:** Isabelle Pierre-Louis, Benita Bamgbade, Sara Lopez-Pintado, Sarah Forrester, Weijia Wang, David McManus, Jane Saczynski

**Affiliations:** University of Massachusetts Medical School, Worcester, Massachusetts, United States; Northeastern University, Boston, Massachusetts, United States; Northeastern University, Boston, Massachusetts, United States; University of Massachusetts Chan Medical School, Worcester, Massachusetts, United States; Johns Hopkins Hospital, Baltimore, Maryland, United States; University of Massachusetts Chan Medical School, Worcester, Worcester, Massachusetts, United States; Northeastern University, Boston, Massachusetts, United States

## Abstract

Although there is a large body of literature linking atrial fibrillation (AF) to adverse clinical outcomes, little is known with regards to the potential links between health-related quality of life (HRQoL) and AF related clinical events. To address this gap, individuals with AF aged ≥ 65 years were recruited from clinics in Massachusetts and Georgia between 2016-18 and followed until 2020. HRQoL was assessed via the Atrial Fibrillation Effect on Quality of Life (AFEQT). The AFEQT is a validated HRQoL measure that quantifies the effect of AF on patients’ daily life on a scale of 0 to 100. AFEQT scores < 80 indicated poor HRQoL and ≥ 80 indicated good HRQoL. The clinical events included major bleeding, stroke, and death. Of the 1,244 participants, the mean age was 75.5 (SD: 7.1), 49% identified as female, 13% were non-White, and 57% were married. After 2 years, 105 major bleeding events (49% poor HRQoL), 19 strokes (42% poor HRQoL) and 108 deaths (59% poor HRQoL) occurred. Our composite outcome included 200 participants (53% poor HRQoL). After adjusting for key covariates, poor HRQoL was associated with an increased risk of our composite outcome (adjusted hazard ratio [AHR]= 1.34, 95% CI: 1.00, 1.80) and death (AHR= 1.58, 95% CI: 1.05, 2.38). We found no significant differences between poor and good HRQoL in major bleeding and strokes. HRQoL is important when assessing health of AF patients. Health providers who treat patients with AF should consider HRQoL when evaluating the risk of death and other adverse outcomes.

